# Preface

**DOI:** 10.2188/jea.JE20101001

**Published:** 2010-05-05

**Authors:** Hirotsugu Ueshima

NIPPON DATA is a prospective observation study named “National Integrated Project for Prospective Observation of Non-communicable Disease And its Trends in the Aged”, and it consists of two collaborators: NIPPON DATA80 and 90. The first follow-up was carried out in 1994 for NIPPON DATA80 and in 1995 for NIPPON DATA90, and both of them are followed up to the present time. The data set is unique due to its representativeness of Japanese people with randomly selected participants numbering around 10 and 8 thousand for NIPPON DATA80 and NIPPON DATA90, respectively. During the past 15 years, the NIPPON DATA80/90 Research Group has published work on many important topics not only for conventional risk factors, but also for current issues such as metabolic syndrome, risk factor clustering, and factors relating to the decline of activity in daily living. They have also published risk assessment charts for myocardial infarction, stroke, and all cardiovascular diseases. These are used in guidelines for the prevention of atherosclerotic diseases, and are now also used as patient education tools.

NIPPON DATA80/90 Nutrition Research is now facing the second phase of NIPPON DATA80/90. It is epoch making that the nutrition and dietary data of the National Nutrition and Health Survey in Japan has been merged with NIPPON DATA80/90. The previous NIPPON DATA80/90 data sets had only individual data of dietary intake obtained from food frequency questionnaires. Therefore, if NIPPON DATA80/90 could also integrate dietary data, a new era would be born because risk assessment would be possible from the points of food and nutrient intake.

In this special issue, we showed the detailed process of how to combine both of the data files: NIPPON DATA80/90 and its relevant year of nutrition data. Then, its validation of using the method of weighing assignment for individual dietary intake from the whole family of dietary intake has been done in this combined data set. Some basic data analyses on nutrient and food intake have been conducted for better understanding of these two unique and fascinating data sets. After these basic analyses, topics related to nutrient and food intake will be done at the next step. The new data set for NIPPON DATA80/90 Nutrition Research will provide us with many important clues on dietary intake and cardiovascular disease prevention, and also on healthy and sound aging.

Hirotsugu Ueshima, MD, PhD

Guest Editor

